# The Results of Legislation

**Published:** 1879-08

**Authors:** 


### THE RESULTS OF LEGISLATION.

The propriety of enacting laws to regulate the practice of dentistry in the several states of this country has been a subject of discussion for the past fifteen years. But few in the beginning espoused the cause, and many prominent men in the profession opposed any such legislation, and some were disposed to cast derision upon it.

The subject became more and more popular till after a time those who had been opposed, for the most part at least, became silent, and by and by espoused the cause. Now scarcely is any one to be found, in the profession, who is really opposed to such legislation. Ohio was the first to secure the enactment of an efficient law to regulate the practice of dentistry. With its provisions all the readers of the REGISTER are perhaps familiar. This was passed in April, 1866.

About this time New York secured the passage of a law which, however, was devoid of any mandatory or restraining character. "This bill did little else than to organize the profession, and duly establish a State Dental Society."

An effort has been made to obtain the enactment of a law that should exert some wholesome restraint. This effort has been crowned with success. On June 20th the Governor signed the bill that had been passed by the last legislature.

The provisions of the bill are essentially the same as those in force in several other States.

Pennsylvania obtained a good law in 1876.

New Jersey also, about the same time, secured a law similar in its leading features.

In 1878 Kentucky legislature passed a law to regulate the practice of dentistry.

And since April last, Indiana rejoices in a law to regulate the practice of dentistry.

The scope and intent of these laws are about the same in each State, which is the suppression of quackery. But the results may be more specifically stated.

In the first place the fact that the profession has a legal recognition and status is a stimulus to its members. In the second place it elevates the profession in public estimation. Again it stops the wholesale production of incompetents—two to six mon th dentists—and frees the people in these States of quacks and charlatans, who migrate to those States where liberty abounds.

Again these laws impel men seeking to enter the profession to gain such an education and attainments as shall enable them to properly meet the reasonable expectations and demands that will be made upon them.

To gain this education they are compelled to resort to the institutions established for the purpose.

This influence is exerted to a far greater extent upon the students, in the States, where these laws are in operation.

In very few instances can a student enter a private office, with a hope of making thorough preparation for practice, in any reasonable time.

Not one practitioner in a hundred has the time, inclination or ability, to properly instruct a student for dental practice. So that, in all States where this law exists, the only resort of the student for a proper preparation for his professional work is the College, and that this is being recognized is shown by the fact that there is a far larger per cent. of students from those States that have dental laws than from those that have not.

The importance of this, to the profession and to the public can not be over estimated.

These facts show, to some extent at least, the responsibility that rests upon our Dental Colleges

